# Moral and Sexual Disgust Suppress Sexual Risk Behaviors among Men Who Have Sex with Men in China

**DOI:** 10.3389/fpsyg.2016.02045

**Published:** 2017-01-09

**Authors:** Jing Zhang, Lijun Zheng, Yong Zheng

**Affiliations:** ^1^Key Laboratory of Cognition and Personality, Ministry of Education, Southwest UniversityChongqing, China; ^2^Faculty of Psychology, Southwest UniversityChongqing, China

**Keywords:** sexual disgust sensitivity, moral disgust sensitivity, sexual risk behaviors, avoidance, men who have sex with men (MSM)

## Abstract

Increasingly more men who have sex with men (MSM) are engaging in sexual risk taking in China in recent years. Given the high rates of HIV infection among MSM in China, it is urgent that we understand the factors that influence MSM's practice of sexual risk taking. Disgust sensitivity, which elicits a behavioral avoidance response, has the potential to influence risky sexual behavior. The present study examined the relationship between disgust sensitivity and sexual risk behavior among MSM in China. Men (*n* = 584) who reported having anal intercourse in the previous 6 months were recruited from the Internet. Two indicators of sexual risk behaviors were measured: condom use and the number of sex partners. The results indicated that moral disgust was positively associated with condom use, with MSM who had higher moral disgust being more likely to use condoms than others did. Sexual disgust was positively associated with the number of sex partners, with MSM who had higher sexual disgust having fewer male sex partners than others did. Sexual and moral disgust sensitivity significantly predicted HIV testing. Our study verified that sexual and moral disgust suppressed sexual risk behaviors and promoted HIV testing. Moral and sexual education should be incorporated in future strategies for HIV prevention and encouragement of safe sex behaviors among MSM in China.

## Introduction

### Sexual risk behavior among MSM in China

The proportion of men infected with HIV/AIDS is growing rapidly among men who have sex with men (MSM) in China. Moreover, unprotected intercourse among homosexuals has gradually become the main transmission route of HIV in recent years. For example, a national surveillance found that HIV prevalence increased from 0.9% in 2003 to 7.3% in 2013 among Chinese MSM, whereas the proportion of HIV infection cases transmitted through sex increased from 2.5% in 2006 to 13% in 2011, and only in 2014 same-sex HIV transmission accounted for 25% among newly diagnosed cases (NSRH, 2014). These data showed that the number of HIV infections related to risky sexual behavior among MSM in China have increased rapidly on an annual basis, as has the likelihood of new infections among MSM due to high-risk sexual behaviors (Liao et al., 2011; Zhou et al., 2013; Shuper et al., 2014).

Given the high rates of HIV infection among MSM in China, it is urgent to understand the factors influencing MSM to engage in sexual risk taking (Fenton and Imrie, 2005; Sullivan et al., 2009). Multiple casual sexual partners and unprotected anal intercourse (UAI) are two major sources of HIV in MSM (Smolenski et al., 2009; Jeffries et al., 2013). Compulsive sexual behavior was positively associated with HIV risk behaviors (Miner et al., 2007), which means that MSM with more compulsive sexual behaviors tend to have more sex partners and high rates of unprotected anal intercourse (UAI, Benotsch et al., 1999). Other studies indicated that MSM who seek sexual partners through the internet have more casual partners and probably engage more frequently in UAI (Benotsch et al., 2002). In China, whose culture is strongly influenced by Confucianism and whose government has given a profound lack of attention to MSM, internalized homophobia (Choi et al., 2008) and a lack of coping skills (Liao et al., 2011) are particularly strong factors correlate with UAI with casual sex partners, and thereby increase the probability of risky sexual behavior.

### Potential relationship between disgust sensitivity and sexual risk behavior among MSM

Disgust sensitivity is a kind of special emotion related to sexual risk behaviors (Bancroft et al., 2003). Fessler and Navarrete (2003) noted that disgust sensitivity has the potential to “reduce participation in biologically suboptimal sexual behaviors” (p. 406). When exposed to abhorrent stimuli, disgust sensitivity protects individuals from being contaminated by harmful substances (Ekman, 1970), which means that disgust sensitivity is linked to the avoidance of infectious situations or objects. For example, individuals with a high level of disgust sensitivity usually exhibit less aggressive behavior (Pond et al., 2012), health anxiety (Thorpe et al., 2003), and are more likely to avoid factors and behaviors related to health threats (Fan and Olatunji, 2013). In fact, disgust sensitivity is as an elicitor of behavioral avoidance, including disease avoidance, and non-normative social behavioral avoidance (Navarrete and Fessler, 2006). According to the disease-avoidance model, heightened vigilance and staying away from serious diseases are two characteristics of individuals who are more sensitive to disgust (Matchett and Davey, 1991; Olatunji et al., 2009). When exposed to stimuli that threaten their health, individuals with a high level of disgust sensitivity usually exaggerate the possibility and severity of being contaminated by such stimuli, which leads to further avoidance. Previous studies have suggested that disgust sensitivity was indeed correlated with avoidance behaviors related to health concerns (Fan and Olatunji, 2013). Logically, engaging in more sexual risk behaviors will result in a greater likelihood of HIV infection; however, HIV is regarded as a serious disease that everyone tries to avoid. Therefore, in the case of HIV infection, MSM with high disgust sensitivity might engage in fewer unsafe sexual behaviors, such as having casual sex with multiple partners.

Theoretically, conservatives have higher disgust sensitivity (Inbar et al., 2009), which is related to their more socially conservative beliefs on topics such as euthanasia, immigration, and abortion (Terrizzi et al., 2010). Disgust sensitivity is also positively correlated with attitudes about upholding traditional sexual morals (Crawford et al., 2014). Other studies also support this viewpoint. For example, individuals with high levels of disgust sensitivity tend to think negatively about sexually licentious behavior, such as having casual sex (Koleva et al., 2012). Consequently, individuals with high levels of sexual disgust tend to dislike casual sex, multiple sex partners, and short time intervals between sexual intercourse; they are also less likely to have sex with potential mates whose health status and personal hygiene are unknown.

Disgust sensitivity has been classified into three types: pathogen, sexual, and moral disgust (Tybur et al., 2009). Individuals' short-term mating strategies (i.e., having sex with multiple partners) usually correlate with low levels of sexual disgust in heterosexuals, and this relationship is independent of individuals' level of pathogen or moral disgust (Al-Shawaf et al., 2015). Short-mating strategies are characterized by short time intervals between sexual intercourse, and the desire for sexual variety increases sexually transmitted infections (Buss, 2012). Based on this, we assumed that MSM in China who have high levels of sexual disgust will use fewer short-term mating strategies, consequently decreasing the rates of risky sexual behaviors.

### The current study

As described above, disgust sensitivity may be a potential factor related to the sexual risk behavior of MSM in China. Individuals with high disgust sensitivity tend to avoid health-related threats and high-risk sexual behavior; they usually have regular partners and more safe sex, which should decrease sexual risk behavior. Therefore, it is important to document the relationship between disgust and sexual risk behavior in an MSM sample in China. Based on the above information and previous studies, we hypothesized that disgust sensitivity would be an important predictor of the suppression of sexual risk behavior.

## Methods

### Participants

This study was conducted online via a Chinese survey website (www.sojump.com). Participants were consisted of 584 MSM of China recruited anonymously from Chinese websites which mainly catering to gay individuals, such as gay forums, gay community and QQ group. All the participants aged from 16 to 56 years (*M* = 21.6, *SD* = 5.4). Demographic characteristics of participants were presented in Table 1. Participants who reported no sexual behaviors were excluded.

**Table 1:** **Sample characteristics and sexual risk behavior condition of MSM in China**.

**Variable**	***N***	**%**
**AGE GROUP**		
16–19	249	42.6
20–25	233	39.9
26–30	74	12.7
≥31	28	4.8
**EDUCATION LEVEL**		
Junior high or less	84	14.4
High school	197	33.7
College or beyond	303	51.9
**MARITAL STATUS**		
Single	450	77.1
Married	31	5.3
Have boy friend	120	20.5
Have girl friend	11	1.9
Divorced	1	0.2
**SEX ROLE**		
Receptive	292	58.3
Versatile	146	29.1
Insertive	63	12.6
**EMPLOYMENT**		
Students	413	70.7
Full time	124	21.2
Unemployment	19	3.3
Others	28	4.8
**MONTHLY INCOME (CNY)**		
0	376	64.4
<2000	58	9.9
2000–4000	87	14.9
4000–6000	33	5.7
6000–10,000	18	3.7
>10,000	12	2.1
Number of general male partners	584	
0	309	52.9
1	116	19.9
2	51	8.7
3+	108	18.5
Number of casual partners	584	
0	388	66.4
1	74	12.7
2	35	6
3+	87	14.9
Condom use as insertive	263	
Never	41	15.6
A few	29	11
Once	32	12.2
Most	64	24.3
Every	97	36.9
Condom use as receptive	285	
Never	39	13.7
A few	21	7.4
Once	31	10.9
Most	80	28.1
Every	114	40
Condom use with MBs partners	100	
Never	19	19
A few	5	5
Once	5	5
Most	11	11
Every	60	60
Condom use with ONS partners	200	
Never	19	9.5
A few	3	1.5
Once	13	6.5
Most	36	18
Every	129	64.5
HIV test	584	
Regular	26	4.5
Once	73	12.5
Never	485	83
HIV status	584	
Negative	133	22.8
Positive	11	1.9
Other	440	75.3

*Men who reported no sexual risk behaviors were exclude. MBs, money boys; ONS, one night stand*.

### Procedure

The online questionnaire consists of three aspects: demographic information, disgust sensitivity, and sexual risk behavior items. Participants first completed the personal basic information of the questionnaire, mainly including age, sex orientation, occupation, and income, and then completed the Three Domain of Disgust Scale. After that, sexual risk behavior section which consists of the items about number of sex partners and condom use.

### Measures

#### Disgust sensitivity

Three Domain of Disgust Scale was designed to measure participants' disgust sensitivity which including 21 items (Tybur et al., 2009). This scale consists of three seven-item subscales: pathogen disgust (e.g., Seeing a cockroach run across the floor), sexual disgust (e.g., Hearing two strangers having sex) and moral disgust (e.g., Deceiving a friend). Two items of sexual disgust sensitivity were not suitable for measuring gay male partners and one item of sexual disgust sensitivity can't be applied for Chinese after factor analysis, so these three items were deleted from the scale. A principal components factor analysis showed that all remaining items loaded as expected based on the original findings (the factor loadings are available from the corresponding author). These three factors accounted for 38.66% of the total variance. Participants should respond how disgusting the situation or act every item described was on a 7-point Likert-type scale (0 = not at all disgusting, 6 = extremely disgusting). The disgust sensitivity scores were defined as the mean score of each disgust domain. The internal consistency reliability (Cronbach's alphas) of the pathogen disgust, sexual disgust, and moral disgust subscales were 0.65, 0.66, and 0.71 respectively.

#### Sexual risk behaviors

The indicators of sexual risk behavior incorporated the following two aspects. Firstly, we measured the number of sex partners in the past 6 month by asking them two items: “how many partners did you have anal intercourse with?,” and “how many men did you have casual sex (one night stand, ONS) with?” (0, 1, 2, 3, 4, 5, 6–7, 8–10, 11–20, more than 21). Secondly, condoms use conditions during the past 6 month were assessed by asking “how often did you use a male condom when you have insertive or receptive anal intercourse with a male partner?,” and “how often did you use a male condom when you have casual sex (ONS) with male partners, when you have anal intercourse with money boys (MBs) partners?” (never = 1, seldom = 2, sometimes = 3, often = 4, always = 5, no such sex = 6). Briefly, we mainly assessed the number of casual sex partners and condom use condition.

#### HIV test and HIV status

HIV test mainly measured the “HIV test condition” (regular, sometimes, never) and “HIV status” (negative, positive, unsure) of MSM during the past 6 months.

## Results

The original choices of participants for each item were shown in Table 1. The mean number of general sex partners was 2.35 (SD = 2.17), 47.1% of the 584 participants had one or more sexual partners. The mean number of casual sex partners was 2.04 (SD = 2.04), 33.6% of the participants had one or more casual sex partners. The proportion of never use a male condom as insertive and as receptive was 15.6 and 13.7% respectively.

### Disgust sensitivity and the number of sexual partners

The correlations among variables were shown in Table 2. Sexual disgust sensitivity was evidently negatively correlated with the number of general male partners (*r* = −0.20, *p* < 0.001), and the number of casual male partners (*r* = −0.18, *p* < 0.001).

**Table 2:** **Correlations between disgust sensitivity and sexual risk behavior**.

**Items**	**Pathogen disgust**	**Sexual disgust**	**Moral disgust**
Number of general male partners (*n* = 584)	0.096^*^	−0.198^**^	0.027
Number of ONS male partners (*n* = 584)	0.082^*^	−0.176^**^	0.020
Condom use as insertive (*n* = 263)	0.009	0.061	0.142^*^
Condom use as receptive (*n* = 285)	0.077	−0.050	0.124^*^
Condom use with MBs partners (*n* = 100)	−0.005	0.060	0.267^**^
Condom use with ONS partners (*n* = 200)	−0.045	0.015	0.153^*^

*Men who reported no sexual risk behaviors were exclude*.

* *p < 0.05*,

** *p < 0.01. MBs, money boys; ONS, one night stand*.

We investigated the relationship between the number of sexual partners and the three domains of disgust sensitivity using multiple linear regression with the items of sexual partners' number as the dependent variable, three disgust sensitivities as the predictors. The results of multiple regressions were shown in Table 3. The results revealed that sexual disgust (*t* = −5.59, β = −0.39, *p* < 0.001) significantly predicted the number of general male partners during the past 6 month, while moral disgust did not predict it. The multiple linear regression model was significant, *F*_(3, 580)_ = 12.30, *R*^2^ = 0.06, *p* < 0.001. Participants' score on the sexual disgust (*t* = −4.90, β = −0.33, *p* < 0.001) evidently predicted the number of casual male partners. The multiple linear regression model was also significant, *F*_(3, 580)_ = 9.40, *R*^2^ = 0.04, *p* < 0.001.

**Table 3:** **Multiple linear regression between disgust sensitivity and sexual risk behaviors**.

	**Pathogen disgust**			**Sexual disgust**			**Moral disgust**					
	**β**	***t***	***p***	**β**	***t***	***p***	**β**	***t***	***p***	***F***	***R*^2^**	***p***
Number of general male partners	0.25	3.08^**^	0.002	−0.39	−5.59^**^	0.000	0.90	0.87	0.384	12.30^**^	0.06	0.000
Number of ONS male partners	0.20	2.69^**^	0.007	−0.33	−4.90^**^	0.000	0.06	0.62	0.534	9.40^**^	0.04	0.000
Condom use as insertive	−0.06	−0.68	0.498	0.04	0.46	0.640	0.24	2.23^*^	0.026	1.99	0.01	0.116
Condom use as receptive	0.05	0.69	0.490	−0.05	−0.76	0.449	0.18	1.85	0.065	1.77	0.01	0.153
Condom use with MBs partners	−0.18	−1.29	0.202	0.03	0.25	0.805	0.52	2.95^**^	0.004	3.04^*^	0.06	0.033
Condom use with ONS partners	−0.13	−1.56	0.120	−0.01	−0.02	0.988	0.27	2.60^**^	0.010	2.43	0.02	0.066

*Men who reported no sexual risk behaviors were exclude*.

* *p < 0.05*,

** *p < 0.01. MBs, money boys; ONS, one night stand*.

### Disgust sensitivity and condom use

We tested whether MSM with greater disgust sensitivity tend to have high rates of condom use. When asking about “how often did you use a male condom when you have insertive anal intercourse with a male partner?” There was significant positive association between “condom use as insertive” and moral disgust sensitivity (*r* = 0.142, *p* < 0.05), but independent of pathogen disgust and sexual disgust. When asking about “how often did you use a male condom when you had receptive anal intercourse with a male partner?” The same as above, “condom use as receptive” was significantly correlated with moral disgust (*r* = 0.124, *p* < 0.05), but not with pathogen disgust and sexual disgust. Moreover, condom use under the condition of having sex with MBs (*r* = 0.267, *p* < 0.01) and casual sex with casual male partners (*r* = 0.153, *p* < 0.05) were both evidently correlated with moral disgust, instead of pathogen and sexual disgust.

The multiple regression with three domain disgust sensitivity as independent variables and condom use condition as dependent variables indicated that only moral disgust sensitivity significantly positively predicted the condition of condom use as insertive (*t* = 2.23, β = 0.24, *p* < 0.05), the condition of condom use with MB partners (*t* = 2.95,β = 0.52, *p* < 0.01) and the condition of condom use with casual partners (*t* = 2.60, β = 0.27, *p* < 0.05), while did not predict the condition of condom use as receptive with the marginal significant(*t* = 1.85, β = 0.18, *p* = 0.065). However, the scores of pathogen disgust and sexual disgust did not predict any items of the condom use condition.

### Disgust sensitivity and HIV test/status

We conducted multiple logistic regression analyses in which HIV status and HIV test respectively was entered as the dependent variable and three domains of disgust sensitivity were entered simultaneously as predictors. The results of Logistic Regression were shown in Table 4. Results found that compared with men whose HIV status was unclear, HIV-positive (OR = 0.46, 95%CI.26-0.80) and HIV-negative men (OR = 0.79, 95%CI.68-0.94) usually were more likely in high sexual disgust sensitivity, while the score on pathogen and moral disgust were not associated with HIV status. Moreover, compared with participants who did not have HIV test, the participants who had HIV test were more likely in higher sexual disgust sensitivity (OR = 1.43, 95%CI 1.19-1.72) and moral disgust sensitivity (OR = 0.75, 95%CI.58-0.99), but there was no significant difference in pathogen disgust (OR = 1.01, 95%CI.83-1.23).

**Table 4:** **Logistic regression between disgust sensitivity and HIV test and status**.

	**Pathogen disgust**			**Sexual disgust**			**Moral disgust**		
	***p***	**OR**	**95%CI**	***P***	**OR**	**95%CI**	***p***	**OR**	**95%CI**
**HIV STATUS**									
HIV-negative	0.938	0.99	0.83–1.19	0.006	0.79	0.68–0.94	0.925	0.99	0.79–1.24
HIV-positive	0.110	1.62	0.90–2.91	0.006	0.46	0.26–0.80	0.816	1.1	0.50–2.39
unclear		1			1			1	
**HIV TEST**									
Yes	0.919	1.01	0.83–1.23	0.000	1.43	1.79–1.72	0.040	0.75	0.58–0.99
No		1			1			1	

## Discussion

The current study found associations among sexual risk behavior, HIV infection, and disgust sensitivity in this Chinese sample of MSM. Of the total number of participants, 47.1% had one or more sexual partners. Additionally, only 36.9% used a condom every time they had insertive anal intercourse with sex partners, and 40% used a condom every time they had receptive anal intercourse. In general, these results were similar to other studies of Chinese gay men (Tang et al., 2013; Zhou et al., 2013). Compared with western countries, it is possible that the prevalence of HIV in China is still not high; however, sexual risk behavior is commonly practiced among MSM and there is an urgent need to address this problem.

In this study, we investigated the factors influencing the sexual risk behavior of MSM in China from a new perspective. As expected, we found that disgust sensitivity was indeed a potential predictor of sexual risk behavior in MSM: MSM with high sexual disgust had fewer sexual partners and MSM with greater moral disgust tended to use condoms more frequently when they engaged in anal intercourse. Thus, our findings on the relationship between disgust sensitivity and MSM's sexual risk behavior support the results of previous studies (Liao et al., 2011; Zhou et al., 2013) and present new evidence for the role of disgust sensitivity in the sexual risk behavior of MSM in China. Moreover, these findings extend the knowledge gained from previous studies related to disgust sensitivity.

Two indicators of sexual risk behaviors among MSM were measured in the present study: casual sex partners and condom use. Each domain of disgust sensitivity had a unique effect on the different indicators of sexual risk behavior of MSM when specific cues activated the three domains. We found that regardless of the situation, the number of sex male partners was negatively correlated with sexual disgust, which was in line with our primary expectation. This result was also consistent with Al-Shawaf's study that found that individuals who preferred short-term mating tended to exhibit low levels of sexual disgust (Al-Shawaf et al., 2015). Others have also reported that sex-related disgust was associated with risky sexual behavior (Stevenson et al., 2011); thus, having multiple sexual partners should increase the likelihood of sexually transmitted infection and trigger cues related to sexual disgust. Therefore, suppressed level of sexual disgust sensitivity is likely to play an important role in the behavior of men's multiple sexual partners.

Additionally, the present results revealed that pathogen disgust sensitivity was positively correlated with the number of male casual sex partners. Previous studies have indicated an association between disgust and sexual arousal (de Jong et al., 2010; Stevenson et al., 2011; Borg and de Jong, 2012; Lee et al., 2014; Grauvogl et al., 2015). Among men, high levels of trait pathogen disgust sensitivity predicted higher levels of genital and subjective sexual arousal (Lee et al., 2014; Grauvogl et al., 2015). It seems that MSM individuals with high levels of pathogen disgust sensitivity are more likely to be sexually aroused and thus will have more casual sexual partners to release sexual arousal. The findings suggest that pathogen disgust does not suppress sexual risk behavior; thus, it does not seem suitable to target pathogen disgust as a means of reducing sexual risk behavior.

The scores on the condom use items were all associated with moral disgust, but independent of pathogen or sexual disgust. The evidence of a correlation between condom use and moral disgust suggests that, relative to “sex,” Chinese MSM may regard condom use as a moral problem. Using a condom when engaged in anal intercourse with another male partner is the most common protective measure for preventing venereal disease. Moreover, this behavior also signals mutual respect between partners for one another in China. Additionally, individuals with high disgust sensitivity tend to be more conservative (Terrizzi et al., 2010) and strongly hold traditional sexual morality (Crawford et al., 2014). According to traditional sexual morals, spiritual and bodily purity are valued (Koleva et al., 2012), and using condoms during anal sex with different sex partners guarantees body purity. Hence, a man who has a strong positive attitude about traditional sexual morality and norms would be more likely to use a condom during anal intercourse with different sex partners. Moreover, the positive association between moral disgust and condom use with MBs might also be interpreted in the context of the “money boys” profession. Money boys are a subgroup of MSM who commercially sell sex to men for economic reason, meanwhile they are the potential group to spread HIV (He et al., 2007; Meng et al., 2010). While they are considered as seriously disgraceful and unethical by others in China. Although different indicators of sexual risk behaviors were correlated with the different domains of disgust sensitivity, the final results of our study support our a priori hypothesis that MSM with greater disgust sensitivity will be less likely to engage in sexual risk behaviors.

Internalized homophobia is another key factor contributing to risky sexual behaviors for MSM in China. Chinese sexual minorities often feel marginalized, experiencing multiple forms of social discrimination, and negative attitudes from multiple parties, including their families, colleagues, and employers (Liu and Choi, 2006). Chinese gay and bisexual men today report greater internalized homonegativity compared to American gay men in 2000 (Zheng and Zheng, 2016). Previous studies have indicated that experiences of homophobia are positively associated with UAI with male partners among MSM (Choi et al., 2008). In the present study, compared with western countries, Chinese MSM experienced more homophobia and discrimination, and thus the greater sexual risk behaviors in this group can be interpreted as being partly due to these two factors.

Sexual and moral disgust were both related to HIV testing—that is, MSM with higher moral and sexual disgust were more likely to obtain an HIV test. This suggests that MSM with higher moral and sexual disgust are more health-concerned, which can promote HIV testing, an avenue toward reducing HIV prevalence. One impact of the effect of disgust on HIV testing is that MSM who knew of their HIV status (negative or positive) had a higher sexual disgust than did others. Despite sexual and moral disgust suppressed risky sexual behaviors and promoted HIV test, moral disgust did not show effect on HIV status.

Disgust sensitivity is an adaptive emotion formed by natural selection under the pressure for human survival. As a kind of typical negative emotion, disgust sensitivity is closely related to disease (Oaten et al., 2009). Therefore, neither moral disgust nor sexual disgust themselves are suitable targets of intervention for reducing sexual risk behaviors of MSM. However, educational interventions could be conducted based on the implications of these types of disgust.

In our present study, moral disgust was positively correlated with condom use condition. The findings highlighted the importance of moral reasoning and responsibility in intervention of HIV among MSM in China. Previous studies indicated that moral reasoning was significantly inversely associated with risk taking during sexual intercourse (Hubbs-Tait and Garmon, 1995), with high moral reasoners acting well both on their intentions to practice less risky behaviors and actual effective behaviors. Furthermore, AIDS knowledge and information could promote safer sexual behaviors only among high moral reasoners, but not among low moral reasoners (Hubbs-Tait and Garmon, 1995). Therefore, the low moral reasoners could be the targeted population in HIV intervention among MSM. Moral development training programs with AIDS information would be a more realistic approach (Hubbs-Tait and Garmon, 1995). Moral reasoning training referred to mainly though labor-intensive discussion of moral dilemmas to foster cognitive conflict and resolved the cognitive dissonance, achieving to a higher level of reasoning (Arbuthnot and Gordon, 1986). This moral reasoning intervention had been documented to be an effective method to suppress sexual risk behaviors recent years (Adam and Husbands, 2008; Cristian Rangel and Adam, 2014). However, less literature currently reported the moral reasoning training in intervening sexual risky behavior (e.g., Cristian Rangel and Adam, 2014), so future studies should pay more attention to the moral reasoning intervention among MSM.

In addition, rational and individual responsibility which assumed that responsibility and morality bounded together and individuals should responsible for their own and partners' health to protect themselves was proved to be effective intervention method (Flowers et al., 2000; Hunt, 2003; Cristian Rangel and Adam, 2014). Individuals' sexual responsibility was related to risk management (Flowers et al., 2000). HIV risk management such as increasing acculturation to gay communities (Kippax et al., 1995), establishing safer sex as a cultural practice (Watney, 1999), knowing HIV transmission routes (Carricaburu and Pierret, 1995) were also important to protect safer sex.

### Limitations and future directions

Despite the findings of our study, it also has some limitations. First, because all of the measures relied on participants' ability to recall their experiences during the past 6 months, recall bias was undoubtedly present when they reported them. Second, we merely investigated the association between disgust sensitivity and sexual risk behaviors in MSM, so future studies should examine the relevance of other influencing factors of sexual risk behaviors, such as sexual arousal, sensation seeking, attitudes toward condom use, or substance use, to disgust sensitivity. Finally, the majority of participants were students recruited from the Internet, which might have resulted in selection bias. Moreover, because most MSM encounter high levels of stigma and prejudice, they often avoid reporting their unsafe sex experiences.

## Ethics statement

Ethics Committee of Southwest University written informed consent was obtained from all participants. All participants were informed that their participation was completely voluntary and that they may withdraw from the study any time. All participants were over 16 years old. Our study is just a questionnaire experiment, which has no injury to all the participants.

## Author contributions

JZ carried out the experimental work and the data collection, interpretation and drafted the manuscript. LZ, JZ, YZ designed the study, interpreted the results, and provided critical review and revision of the article.

### Conflict of interest statement

The authors declare that the research was conducted in the absence of any commercial or financial relationships that could be construed as a potential conflict of interest.
